# Special Issue “Role of Exercises in Musculoskeletal Disorders—4th Edition”

**DOI:** 10.3390/jfmk10010069

**Published:** 2025-02-19

**Authors:** Giuseppe Musumeci

**Affiliations:** 1 Department of Biomedical and Biotechnological Sciences, Section of Anatomy, Histology and Movement Science, School of Medicine, University of Catania, 95123 Catania, Italy; giuseppe.musumeci@unict.it; 2 Research Center on Motor Activities (CRAM), University of Catania, 95123 Catania, Italy

## 1. Introduction

This fourth edition of the Special Issue titled “Role of Exercises in Musculoskeletal Disorders” significantly enriches the discourse on the effectiveness and feasibility of physical activity in managing patients with musculoskeletal disorders. It presents a variety of studies—from reviews to original investigations—with the aim of exploring the effects of physical activity on people with these conditions.

The positive effects of exercise on musculoskeletal function, quality of life and a huge variety of health outcomes are well known and extensively reported in the literature [1]. However, research on kinesiology applied to musculoskeletal disorders continually introduces new exercise modalities and therapeutic approaches aimed at improving patient outcomes and expanding the horizons of care. Physical activity has proven to be effective in ameliorating the quality of life, autonomy and symptoms of patients affected by both musculoskeletal and cognitive disorders [2,3,4,5]. Given the complex nature of musculoskeletal disorders and the individualized needs of patients, the development and dissemination of scientifically validated exercise interventions are crucial for both practitioners and researchers. A comprehensive understanding of the advantages and limitations of these methods is essential to effectively manage, rehabilitate, and enhance the autonomy of patients with various conditions, from chronic low back pain to tendinopathies and age-related musculoskeletal decline. This Special Issue addresses these critical gaps with a variety of studies, ranging from narrative and systematic reviews to original investigations, aiming to deepen our understanding of the effects of physical activity on individuals with musculoskeletal disorders. The research included covers topics such as the reliability of muscle ultrasonography, intergenerational physical activity programs, low-molecular-weight hyaluronic acid treatments, ergonomics in cycling, core stability exercises, and metabolic supplementation with L-carnitine. By examining multifaceted approaches, this Special Issue seeks to provide a deeper comprehension of how tailored exercise programs can optimize musculoskeletal health and rehabilitation outcomes.

## 2. Overview of Published Articles

Understanding the effects of different types of exercise intervention, as well as the potentiality and limitations of technologies in acquiring reliable data, is fundamental in improving knowledge in this field of research. Carr et al. [6] focused on the reliability of skeletal muscle ultrasonography when performed by novice sonographers; they found that while novice sonographers could reliably acquire muscle size measurements with minimal training, muscle quality measurements, such as echo intensity, required more experience for consistent results. Another area of interest investigated was the promotion of physical activity among different age groups to improve social spheres. Novak and colleagues [7] highlighted how the method of determining maximal voluntary contraction (MVC) can influence the force control strategies, especially at high intensities. They found that the sustained group demonstrated greater precision and complexity in force fluctuations, while the discrete group relied on less accurate strategies beyond 65% MVC. The findings challenge the dominance of discrete MVC protocols, emphasizing how sustained methods may better reflect real-world tasks requiring visuo-motor precision and providing valuable information for researchers in this field of study. Wittig et al. [8] investigated anterior cruciate ligament (ACL) reconstruction practices in different countries by analyzing national knee ligament registries from Sweden, the UK, New Zealand, and Norway. Significant differences were observed in reconstruction rates, ranging from 4.1 to 51.3 per 100,000 people, as well as in the delay between injury and surgery, which varied from 6 to 17.6 months. The sports most commonly linked to ACL injuries included soccer, alpine skiing, handball, rugby, and netball, and there was substantial variability in graft choices for reconstruction. With this study, the authors highlighted the value of national registries in improving patient outcomes and surgical quality through real-world data analysis. Aldajah et al. [9] evaluated the effectiveness of extracorporeal shock-wave therapy (ESWT) compared to conventional physiotherapy in treating lateral epicondylitis, dividing their sample in two groups—one receiving ESWT and one performing conventional physiotherapy—with both completing five treatment sessions. Both groups showed significant improvement in pain reduction, grip strength, and upper-extremity function, as measured by the Visual Analog Scale (VAS), Disabilities of the Arm, Shoulder, Hand questionnaire, and a dynamometer. However, the ESWT group demonstrated superior outcomes in all measures, suggesting that five sessions are particularly effective for pain relief and functional improvement in lateral epicondylitis. Buonsenso et al. [10] studied the willingness and enjoyment of elderly individuals participating in physical activities alongside preschool children, revealing that 81.5% of elderly participants expressed positive feelings toward intergenerational physical activity programs, compared to only 44.3% who enjoyed their usual physical activity, suggesting that such programs could effectively combat sedentariness in both age groups by fostering mutual participation and enjoyment. The management of tendinopathies has also seen advancements, particularly with the use of low-molecular-weight hyaluronic acid injections (LMW-HA), as discussed in a narrative review [11] that highlighted how LMW-HA injections can reduce pain and improve tendon function by enhancing collagen synthesis and reducing inflammation. Moreover, Scaturro and colleagues [12] studied the effectiveness of hybrid hyaluronic acid (HA) viscosupplementation compared to high-molecular-weight HA in treating hip osteoarthritis (OA) in overweight and obese patients. Eighty participants were enrolled and divided into two groups: one received two ultrasound-guided injections of hybrid HA, while the other received a single injection of medium-high molecular weight HA. It emerged that the hybrid HA group showed significant improvements in pain reduction, functional capacity, and walking performance, highlighting the superior anti-inflammatory and analgesic properties of hybrid HA and its promising characteristics as a treatment for hip OA in overweight or obese patients. In their systematic review, Chiaramonte et al. [13] investigated diagnosis, rehabilitation, and preventive strategies for cyclists with pudendal neuropathy, a condition that can lead to symptoms such as pain, numbness, and erectile dysfunction. The authors emphasized the importance of proper bicycle ergonomics and preventive strategies to mitigate this condition with adjustments such as using padded, wide saddles and maintaining handlebars at a height parallel to or above the saddle to reduce perineal pressure, providing practical information for cycling practitioners. Another review included in the current Special Issue [14] focused on the efficacy of core stability in non-specific chronic lower back pain, concluding that core stability exercises are highly effective in reducing pain intensity and functional disability, while improving quality of life and core muscle activation. Combining core stability exercises with other therapeutic modalities, such as respiratory training, gluteal muscle strengthening, or neuromuscular electrical stimulation, elicited even greater benefits. Painter et al. [15] studied the relationship between countermovement jump (CMJ) impulse asymmetry and performance in NCAA Division I soccer athletes, addressing inconsistencies in asymmetry assessment methods. Using both unweighted (CMJ0) and weighted (CMJ20) jumps, asymmetry was evaluated through propulsive-phase asymmetry scores (PrPASs) and positive impulse asymmetry scores (PIASs). Significant correlations were observed between jump height and PIASs; unweighted PIASs were negatively correlated with jump height in females, while weighted PIASs showed a similar negative correlation in males. PrPAS metrics did not show significant correlations. The findings suggest that both weighted and unweighted CMJ testing should be used, with PIASs as the preferred metric for assessing asymmetry’s impact on performance. Lee et al. [16] investigated the relationship between the extracellular to intracellular body water ratio and cognitive function across age groups, finding that the former increases with age and predicts cognitive performance, particularly executive function and attention. The ratio demonstrated strong sensitivity (83%) and specificity (91%) in detecting lower executive function scores, suggesting that the extracellular to intracellular body water ratio is a valuable predictor of cognitive function, even in healthy older adults, highlighting its potential as a marker for age-related cognitive changes. In their systematic review, Vecchio et al. [17] evaluated the effects of L-carnitine supplementation on physical performance and metabolic parameters in healthy individuals, aiming to determine the optimal dosage for maximum benefit. It was found that while L-carnitine did not significantly affect serum lactate levels at rest or after exercise, it did improve maximal oxygen consumption (VO2) at rest and significantly increased serum total and free carnitine levels both at rest and after exercise. Effective dosages included 2 g/dL for 4 weeks and 1 g/dL for 3 weeks, depending on the parameter measured. These findings suggest that serum carnitine levels and VO2 at rest are useful markers to monitor during physical activity or rehabilitation programs, emphasizing the importance of tailored supplementation. Lastly, Scaturro et al. [18] explored the impact of high-dose vitamin D supplementation combined with physical exercise on pain, functional capacity, and quality of life in fibromyalgia patients, with an emphasis on age-related differences. Eighty patients were divided into two groups: those aged ≤ 50 years and those > 50 years. Both groups received 50,000 IU of vitamin D weekly for three months alongside a rehabilitation protocol. Interestingly, younger patients experienced short-term pain relief and long-term functional improvement, while older patients showed improvements in long-term quality of life and pain, suggesting that vitamin D supplementation enhances outcomes in fibromyalgia, with benefits varying by age group.

## 3. Conclusions

This fourth edition of the Special Issue “Role of Exercises in Musculoskeletal Disorders” highlights our expanding understanding of physical activity’s impact on musculoskeletal health and rehabilitation. Through diverse studies, this collection underscores the efficacy of tailored exercise programs, complementary therapies such as L-carnitine, and vitamin D supplementation in improving pain, function, and quality of life across various conditions. Innovative approaches like intergenerational physical activity programs, core stability training, and novel evaluation methods enrich the discourse. All the findings contained in this Special Issue emphasize the importance of individualized, evidence-based interventions to address the complex needs of musculoskeletal disorder patients, laying the foundation for future advancements in care and research.
